# Global pleiotropic effects in adaptively evolved *Escherichia coli* lacking CRP reveal molecular mechanisms that define the growth physiology

**DOI:** 10.1098/rsob.210206

**Published:** 2022-02-16

**Authors:** Ankita Pal, Mahesh S. Iyer, Sumana Srinivasan, Aswin Sai Narain Seshasayee, K. V. Venkatesh

**Affiliations:** ^1^ Department of Chemical Engineering, Indian Institute of Technology Bombay, Powai, Mumbai 400076, India; ^2^ National Centre for Biological Sciences, GKVK, Bellary Road, Bangalore 560065, India

**Keywords:** adaptive evolution, cAMP receptor protein, pleiotropic effects, exponential growth, intracellular metabolites

## Abstract

Evolution facilitates emergence of fitter phenotypes by efficient allocation of cellular resources in conjunction with beneficial mutations. However, system-wide pleiotropic effects that redress the perturbations to the apex node of the transcriptional regulatory networks remain unclear. Here, we elucidate that absence of global transcriptional regulator CRP in *Escherichia coli* results in alterations in key metabolic pathways under glucose respiratory conditions, favouring stress- or hedging-related functions over growth-enhancing functions. Further, we disentangle the growth-mediated effects from the CRP regulation-specific effects on these metabolic pathways. We quantitatively illustrate that the loss of CRP perturbs proteome efficiency, as evident from metabolic as well as ribosomal proteome fractions, that corroborated with intracellular metabolite profiles. To address how *E. coli* copes with such systemic defect, we evolved *Δcrp* mutant in the presence of glucose. Besides acquiring mutations in the promoter of glucose transporter *ptsG*, the evolved populations recovered the metabolic pathways to their pre-perturbed state coupled with metabolite re-adjustments, which altogether enabled increased growth. By contrast to *Δcrp* mutant, the evolved strains remodelled their proteome efficiency towards biomass synthesis, albeit at the expense of carbon efficiency. Overall, we comprehensively illustrate the genetic and metabolic basis of pleiotropic effects, fundamental for understanding the growth physiology.

## Graphical abstract

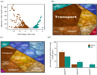

## 1. Introduction

Global transcriptional factors represent a cornerstone in the transcriptional regulatory network (TRN), which facilitates system-wide changes in gene expression levels in response to alterations in its external or internal environment [1–3]. Considering the complex interactions existing within the TRN of an organism, the absence of global transcription factors results in direct or indirect cellular responses that incapacitate the ability to attain favourable phenotypic outcomes, even for a simple prokaryote like *Escherichia coli*. Understanding the regulatory mechanisms of the global transcriptional regulator CRP (cAMP receptor protein) under diverse environmental conditions has been an area of research for many decades. CRP, along with its cognate signalling molecule cAMP [4–6], activates transcription at more than 200 promoters, as evidenced from the genome-wide binding and reporter-based studies in *E. coli* [7–9]. *In vitro* and *in vivo* binding assays have determined and validated the genome-wide binding sites of CRP, along with its interactions with RNA polymerase [10,11]. Several studies have shown that CRP regulates numerous processes such as (i) transport and metabolism of various carbon sources such as glucose, mannose and galactose [9,12–16], (ii) regulation of enzymes of the tricarboxylic acid (TCA) cycle, and oxidative phosphorylation [12,17,18], (iii) stress response and osmoregulation [19–23], (iv) nitrogen and iron assimilation [24–27], (v) stringent response [28], as well as (vi) resistance to multiple antibiotics [29,30]. Moreover, the physiological significance of its activator molecule cAMP, in coordinating the carbon and nitrogen demands via carbon catabolites, addressed the long-standing debate on carbon catabolite repression [31].

Despite the huge repository of data available for CRP, several questions are still unanswered. As changes in carbon transport rate can only in part explain the changes in growth physiology [32,33], synthesis of precursors or biomass components by metabolic enzymes and ribosomes, and their attuned efficiency coordinated by CRP, remain obscure. In addition, delineating the CRP regulation-specific effects on these molecular mechanisms from those mediated by changes in growth rate becomes imperative. Moreover, proteome allocation principles [31,34,35] that facilitate system-wide fine-tuning of the necessary and unnecessary metabolic proteome towards biomass synthesis mediated by CRP deserve attention. Scarce knowledge of the fate of cAMP and downstream metabolite profiles in the absence of CRP limits our ability to link the molecular consequences to such proteome partitioning. Thus, a fundamental question that arises now is, how the interactions within these interdependent factors such as glucose import, proteome allocation, and metabolite adjustments coordinated by CRP facilitate the increased growth rate of an organism.

By exploiting these molecular interactions, we sought to investigate how an *E. coli* K-12 MG1655 strain lacking CRP can cope with this global disruption using adaptive laboratory evolution (ALE) under glucose minimal media conditions. ALE entails the orchestration of genetic as well as phenotypic behaviour in response to mutations that provide growth fitness benefits to organisms under strict selection pressures [36–39]. While a majority of the ALEs have focused on understanding the adaptive rewiring in response to the loss of metabolic genes [40–42], studies that focus on ALEs on the loss of global transcriptional regulators are now emerging [43,44]. Importantly, the global pleiotropic effects of mutations in regulator deleted strains, on cellular proteomic and metabolomic resources that would enable their growth recovery have not been addressed. Therefore, to decipher the underlying molecular basis of divergence of evolved strains away from their ancestor [45–48], examining the evolution of a *crp* mutant with integration of transcriptomics, metabolomics and proteome allocation aspects would be of great value.

In this present study, using a multi-omics approach, we explicitly characterize the physiological significance of CRP for exponential growth in glucose minimal media conditions. We demonstrate the systems-wide pleiotropic effect of beneficial mutations on cellular processes underlying increased growth rate in evolved *E. coli* strains lacking CRP. Further, we elucidate in detail, its underlying direct regulatory or indirect growth-rate-dependent mechanisms that coordinate metabolite profiles and the allocation of proteomic resources towards its cellular objectives. Overall, by evaluating such genotype–phenotype relationships in the parent and evolved strains, we unravel the inherent constraints of genetic and metabolic networks underlying evolvability in *E. coli*.

## 2. Results

### 2.1. Loss of CRP caused large shifts in the transcriptome of key metabolic pathways

**Figure 1.**
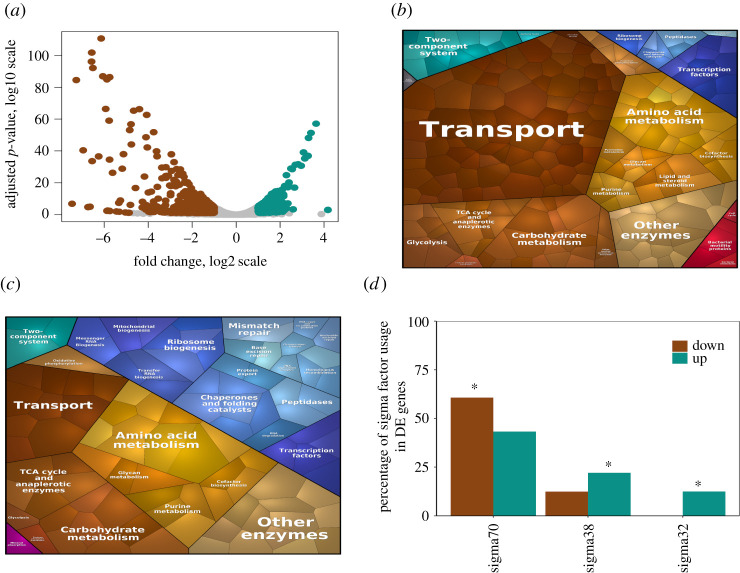
Severe perturbation of gene expression in *Δcrp* compared to WT. (*a*) Volcano plot of the DE genes *Δcrp* compared to WT depicted as adjusted *p*-value (log10 scale) versus fold change (log2 scale). The brown dots indicate downregulated genes and the cyan dots indicate upregulated genes. (*b*) Voronoi treemaps showing the downregulated metabolic pathways enriched by KEGG classification. Transport, carbohydrate metabolism and TCA cycle were found to be significantly downregulated (*p* < 0.05). (*c*) Voronoi treemaps showing the upregulated metabolic pathways enriched by KEGG classification. Chaperone and folding catalysts, other enzymes and TCA cycle were found to be significantly upregulated (*p* < 0.05). The size of the hexagon within each pathway is directly proportional to the absolute fold change observed for the genes. The colour of the hexagon denotes the specific pathways classified by KEGG. (*d*) Enrichment of genes under the regulation of sigma factors; the brown bars and the cyan bars indicate the fraction of downregulated and upregulated genes in *Δcrp* versus to WT respectively. Significant increase or decrease is denoted by asterisks (*p* < 0.01).

CRP occupies an apex node in the hierarchical TRN of *E. coli* regulating a myriad of genes under diverse nutritional conditions [3]. We first addressed the systemic effect caused by the loss of CRP by performing high-coverage RNA sequencing of *Δcrp* mutant in glucose minimal media condition during the mid-exponential phase. The transcriptome of this strain, when compared to its parent wild-type (WT) strain, showed ∼725 differentially expressed (DE) genes (absolute fold change (aFC) ≥ 2, adjusted *p*-value (adj-*p*) less than 0.05) of which ∼534 genes (74%) were downregulated and ∼191 genes (26%) were upregulated in the mutant (figure 1*a*), indicating a large upset of the global transcriptome. This reiterated the role of CRP as a transcriptional activator, which was in good agreement with previous gene expression studies [9,11,18] (electronic supplementary material, file S1).

Next, we examined the KEGG pathways, which were significantly enriched among these DE genes and represented them as Voronoi treemaps (figure 1*b,c*). Out of the total 725 DE genes, 346 genes (242 downregulated and 104 upregulated genes) were enriched for KEGG pathways (electronic supplementary material, file S1). Among these enriched pathways, downregulated genes were significantly associated with transporters, TCA cycle (*sucABCD and sdhABCD*) needed for energy generation during aerobic respiration, and carbohydrate metabolism involved in the processing of secondary carbon compounds such as uronic acid (*garD, kduI, uxaAC, uxuA*), galactitol (*gatDYZ*) and glucan (*malPQS*) metabolism. Downregulation of transporters was associated with the major glucose transporter (*ptsG*), secondary glucose transporters (*manXYZ*, *malEFGKX* and *lamB* that function under glucose limitation), transport of amino acids (*tdcC*, *proVXW*, *hisJ*, *livJKH*, *lysP*, *leuE*) and nucleotides (*tsx*, *uraA*, *nupCGX*), as well as alternate carbon transporters (*glpF*, *fruB*), in agreement with previous studies (electronic supplementary material, file S1). Moreover, we observed the upregulated genes to be significantly associated with other enzymes and, chaperone and folding catalysts. The chaperone genes (*ibpAB*, *hslR*, *htpG*, *cbpA*) are required to maintain proper protein turnover and integrity and their upregulation might indicate a response to the stress encountered by the cell [49]. Similarly, the genes of the other enzymes category were associated with fatty acid metabolic process (*ahr, cfa*), peptidoglycan biosynthesis (*murG, mepA*) and genes expressed in response to stress (*dosP, pphA, katE*). Further, we found upregulated genes enriched in the TCA cycle and anaplerotic enzymes to be primarily involved in glycolate metabolism (*glcDEF*) and glyoxylate degradation (*aceAK*). These genes are unnecessary during glucose metabolism and their upregulation indicates an increase in hedging mechanism related to alternate carbon metabolism [45,50,51]. Overall, the considerable shifts in the transcriptome of these pathways emphasize the metabolic dysregulation caused by the loss of a global regulator.

We identified a significant fraction of KEGG-enriched downregulated genes (approx. 46%, *p* < 10^−26^) that were found to be regulated by CRP, as opposed to the upregulated genes (approx. 10%, *p* > 0.1) using targets identified from the EcoCyc database. Such gene expression patterns could be attributed to the direct and indirect effects of loss of CRP regulation. Moreover, approximately 61% (*p* < 10^−2^) of enriched downregulated genes were found to be regulated by sigma 70, the major growth-related sigma factor associated with RNA polymerase (figure 1*d*), corroborating the association of CRP with sigma 70 reported previously [10,11]. A significant fraction of enriched upregulated genes were regulated by stress-related sigma factors, sigma 38 (approx. 22%, *p* < 10^−4^) and sigma 32 (approx. 13%, *p* < 10^−2^). Presumably, this suggested the reallocation of RNA polymerase away from growth and towards stress-related genes as an indirect consequence of the loss of a global regulator.

**Figure 2.**
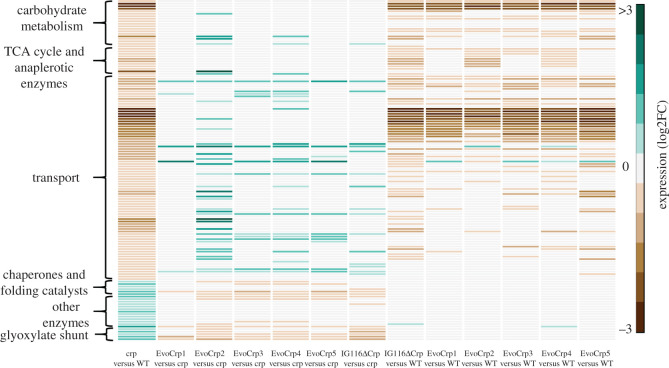
Dissecting the direct effects of CRP and mutation from the indirect effects of changes in growth rates. (*a*) The stacked plots showing the percentage of CRP-specific effects and growth-mediated effects in the genes of the downregulated and upregulated KEGG pathways in *Δcrp* versus WT. (*b*) The stacked plots showing the percentage of mutation-specific effects and growth-mediated effects in the genes of the downregulated and upregulated KEGG pathways in the EvoCrp strains (EvoCrp3 shown in figure) versus *Δcrp*. (*c*) The stacked plots showing the percentage of mutation-specific effects and growth-mediated effects in the genes of the downregulated and upregulated KEGG pathways in the IG116-*Δcrp* strain versus *Δcrp*. Significant pathways are denoted by asterisks (*p* < 0.05).

As growth rate changes [52–54] have a profound effect on gene expression, we sought to disentangle the effects on the DE genes caused directly due to the loss of CRP from these growth-mediated effects. We carried out RNA-sequencing of the WT and the *Δcrp* cultivated in glucose-limited chemostat conditions at a fixed dilution rate of 0.21 h^−1^. First, the key metabolic gene expression changes in glucose-limited chemostat cultivation for *Δcrp* compared to WT were consistent with that observed in glucose excess batch conditions. Further, the genes that were not differentially expressed in the glucose-limited chemostats were attributed to lowered growth rates. Genes that were differentially expressed in *Δcrp* compared to WT under chemostat conditions represent the genes that are specific to CRP regulation or genes that are not altered due to slow growth effects. These genes were used to distinguish the CRP regulation-specific and growth-mediated changes in KEGG pathways observed under batch exponential growth. Overall, we observed approximately 64% of the KEGG enriched upregulated genes (63 out of 104 genes) and downregulated genes (159 out of 242 genes) to be directly regulated by CRP, as opposed to 36% that were due to the slow growth rate mediated effects (figure 2*a*; electronic supplementary material, file S1). Of the pathways found to be downregulated, we found 79% of carbohydrate metabolism genes (15 out of 19 genes), 68% of transport genes (56 out of 82 genes) and 81% of the TCA cycle genes (9 out of 11 genes) enriched due to CRP regulation. Similarly, of the upregulated pathways, genes for other enzymes were found to be under co-regulation of CRP and growth, whereas 83% of genes of chaperones and folding catalysts (5 out of 6 genes) and 80% of TCA cycle genes (4 out of 5 genes) were found to be enriched mainly due to the growth-mediated effects. The KEGG-pathway-enriched DE genes identified to be CRP-specific were compared with known CRP regulated promoters from previous studies and prediction using consensus motif sequence of CRP binding. Indeed, the majority of these genes could be attributed to being direct targets of CRP (electronic supplementary material, file S1). Therefore, these data assert the significant regulation of CRP on several metabolic pathways.

### 2.2. Adaptive evolution involves mutations in the intergenic region of the glucose transporter

To decipher how *Δcrp* copes with the perturbations in its global gene expression, we adaptively evolved five independent replicates of the mutant with multiple passages in batch culture with non-limiting glucose, strictly during the mid-exponential phase (electronic supplementary material, figure S1A). The mutant was evolved until the growth rate showed no further increase in subsequent passages. We observed that the growth defect in *Δcrp* was rapidly recovered (electronic supplementary material, figure S1B) within approximately 100 generations of adaptive evolution and the endpoint populations (EvoCrp) were further characterized in this study (electronic supplementary material, file S1).

We performed whole-genome resequencing (WGS) to identify the causal mutations in all the EvoCrp populations (electronic supplementary material, table S1 and text S1)*.* Out of eleven unique mutations detected across all the EvoCrp strains, six were in the upstream promoter sequence of the *ptsG* gene, a component of the phosphotransferase system (PTS) responsible for ATP-independent glucose uptake in *E. coli*, known to be under the positive regulation of CRP [9,11,18]. Previous studies involving aerobic evolution of the WT in glucose minimal media did not incur mutations in the promoter region of the *ptsG* gene [36,55,56]. This asserted that these mutations were indeed in response to the loss of CRP and not due to adaptation to glucose in the medium. The intergenic mutations were mostly SNPs specifically in the binding sites of the repressors namely Fis [57,58], Mlc [59,60] and ArcA [61], reported to repress *ptsG* gene expression (electronic supplementary material, figure S2). We therefore hypothesized that these mutations in the *ptsG* promoter region are responsible for altered binding affinity, resulting in the de-repression of the *ptsG* gene.

First, to examine the adaptive role of the mutations, we introduced the IG116 promoter mutation (as annotated in electronic supplementary material, table S1) in the *Δcrp* background. The introduction of the mutation resulted in approximately 85% recovery to the WT growth rate, thereby confirming the adaptive nature of the promoter mutations in the EvoCrp strains (electronic supplementary material, figure S3A,B and text S1). Next, to test our altered binding hypothesis, we characterized the *in vivo* binding affinity of Fis and Mlc in IG116-Δ*crp* mutant strain using CHIP-qPCR. We did not observe any significant enrichment for Fis or Mlc binding to the promoter either in the IG116-Δ*crp* strain or in the WT and *Δcrp* strains (electronic supplementary material, figure S4A-D), which was in agreement with a previous study in *E. coli* K-12 MG1655 [58]. This emphasized the fact that the regulation of *ptsG* is not dependent on the interplay of the regulators Fis and Mlc. Also, a detailed analysis of the WGS data indicated that most of the mutations in the *ptsG* promoter resulted in new transcriptional start sites (TSS) with ‘Pribnow’ box-like consensus sequence (electronic supplementary material, text S1), thereby reiterating that these *ptsG* promoter mutations could potentially augment the affinity of RNA Polymerase sigma 70, as previously observed in a recent study, albeit in a different genetic background [43]. However, the mutation profile and the binding profile of negative regulators on the *ptsG* promoter in our study were markedly different from the previous study that can be attributed to the underlying differences in the genetic background of the parent strains used for evolution in both the studies.

### 2.3. Adaptive rewiring of gene expression of metabolic pathways in EvoCrp strains

To understand the gene expression changes that enabled enhanced growth rate of the evolved strains, we characterized the transcriptome of the EvoCrp strains by comparing it to the *Δcrp* parent strain as well as the WT strain (electronic supplementary material, file S1). Broadly, all EvoCrp strains compared to *Δcrp* showed fewer DE genes (ranging from approx. 45 to approx. 350 DE genes) as opposed to approximately 700 DE genes observed in *Δcrp* compared to WT (electronic supplementary material, figure S5A). In addition, a higher proportion of DE genes in EvoCrp showed upregulation relative to *Δcrp* (electronic supplementary material, figure S6A–E)*.* To gain a preliminary understanding of the adaptive response of EvoCrp, we computed the correlation between the fold-change of gene expression in *Δcrp* versus WT and EvoCrp versus WT (electronic supplementary material, figure S7A-E) and observed a strong positive correlation (Pearson correlation coefficient, *r* = 0.74, *p* < 10^−15^). Also, the magnitude of the difference of fold change between EvoCrp and WT was lesser compared to *Δcrp* versus WT (*p* < 10^−15^, Mann–Whitney test). Both of these observations indicated a partial restoration in EvoCrp gene expression states towards WT levels. The transcriptome comparison of the EvoCrp strains to the WT showed approximately 170–390 DE genes (electronic supplementary material, figure S5B) with a large number of genes found to be downregulated compared to the upregulated genes.

Next, we investigated the metabolic pathway enrichment in EvoCrp strains. For instance, across a majority of EvoCrp strains compared to *Δcrp*, we observed upregulated genes significantly associated with transporters, and downregulated genes significantly associated with carbohydrate metabolism (related to osmotic stress), TCA cycle and anaplerotic enzymes (related to hedging mechanisms) and chaperone and folding catalysts (electronic supplementary material, file S1). The restoration in the gene expression in EvoCrp relative to *Δcrp* also suggested a repartitioning of RNA polymerase sigma factors, as indicated by the large fraction of the upregulated genes regulated by RNA polymerase sigma 70 (approx. 76%, *p* < 10^−5^) and the downregulated genes regulated by stress-related sigma factors, sigma 38 (approx. 37%, *p* < 10^−3^) and sigma 32 (approx. 14%, *p* < 10^−13^) (electronic supplementary material figure S8A–E). Overall, in EvoCrp strains, we observed rewiring of metabolic pathways, favouring growth over stress-related functions.

**Figure 3.**
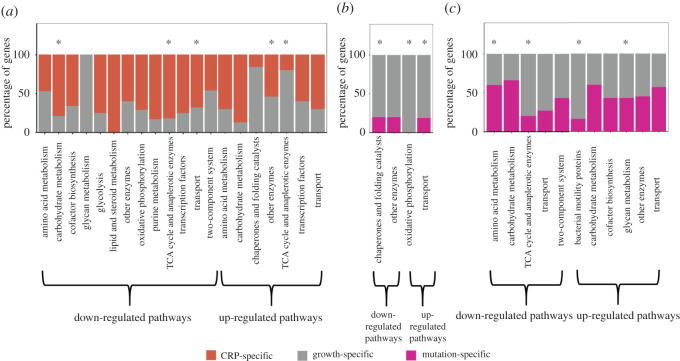
Comparative analysis of the KEGG pathway enriched genes across all the strains. Heatmap depicting the genes of the significantly altered pathways in *Δcrp* compared to WT. These pathways were also analysed in the EvoCrp strains and IG116-*Δcrp* compared to *Δcrp* and WT to identify the pattern of recovery in the gene expression.

Further, KEGG pathway enrichment of the DE genes found in EvoCrp compared to WT indicated significant downregulation of TCA and anaplerotic enzymes (such as *sucABCD* and *sdhABCD*), carbohydrate metabolism (such as *gatDYZ*, *malPQS*), and lipid metabolism (such as *dhaKL*, *fadBI*) (electronic supplementary material, file S1). Additionally, we observed the downregulation of transporter genes (associated with secondary transporters of glucose such as *manXYZ*, *malEFGK*, *lamB*) that are unnecessary for exponential growth on glucose. By contrast, we observed no pathway to be significantly upregulated in EvoCrp strains except for the significant upregulation of amino acid metabolism and purine metabolism only in EvoCrp5. This indicated that the majority of the pathways that were affected in *Δcrp* were reverted in the EvoCrp strains to the pre-perturbed state (figure 3).

To disentangle the effects of increased growth on gene expression from the mutation-specific effects, we performed glucose-limited chemostat cultivations of two of the EvoCrp strains (EvoCrp1 and EvoCrp3) at a dilution rate of 0.21 h^−1^. The DE genes observed in these EvoCrp strains when compared to *Δcrp* grown under chemostat conditions indicated the effects on gene expression mediated due to the *ptsG* promoter mutations acquired during ALE. These genes were then used to determine the growth-specific and mutation-specific effects across all the EvoCrp strains during its batch exponential growth. The mutation-specific effects involved downregulation of unnecessary metabolic genes irrespective of the growth and glucose (excess versus limiting) conditions. Further, changes in growth genes that were not differentially expressed in the glucose-limited chemostats were attributed to increased growth rates. Of the KEGG enriched DE genes, we observed 30–35% changes due to the *ptsG* promoter mutations as opposed to approximately 65–70% as a result of increased growth rate (figure 2*b*; electronic supplementary material, file S1). The mutation-specific effects across the majority of the EvoCrp strains entailed upregulation of genes responsible for glucose uptake (*ptsG*), as well as reduction of unnecessary genes involved in glycolate metabolism and amino acid degradation (electronic supplementary material, file S1).

Next, we asked whether the restoration pattern is relevant even in the case of the introduction of a single point mutation in a *Δcrp* strain (IG116-Δ*crp*). We observed substantial differences in the gene expression patterns of metabolic pathways (IG116-Δ*crp* versus *Δcrp*) compared to the evolved populations (EvoCrp versus *Δcrp*) that emphasized the implication of ALE in facilitating the final phenotypic outcome of the organism (electronic supplementary material, file S1 and text S1). Despite the differences, on determining the genes unperturbed as a result of increased growth rate and that responded purely to the introduced point mutation, IG116-Δ*crp* showed a reduction in unnecessary genes associated with alternate carbon metabolism, amino acid degradation and osmotic stress, akin to the evolved populations (figure 2*c*; electronic supplementary material, file S1). The amino acid degradation genes are associated with the degradation of amino acids that yield ammonia as the end product that can potentially quench the nitrogen requirement of the organism [62]. These genes were found to be upregulated in *Δcrp* mutant compared to WT. As a result of the IG116 mutation, we speculate that the mutant strain was able to overcome the nitrogen deficiency, thereby eliminating the need to generate ammonia by amino acid degradation rendering them unnecessary. Overall, amino acid degradation, alternate carbon metabolism and osmotic stress-related genes represent the unnecessary genes that cause a burden on the proteome, thereby constraining optimal biomass synthesis. As faster growth has an inverse correlation with the expression of unnecessary genes, the IG116 mutation enabled reduction of the allocation of resources towards unnecessary or stress-related genes and increased allocation of resources towards the expression of the necessary growth-related genes, similar to the EvoCrp strains.

### 2.4. Accumulation of metabolites illustrates strain-specific growth effects

**Figure 4.**
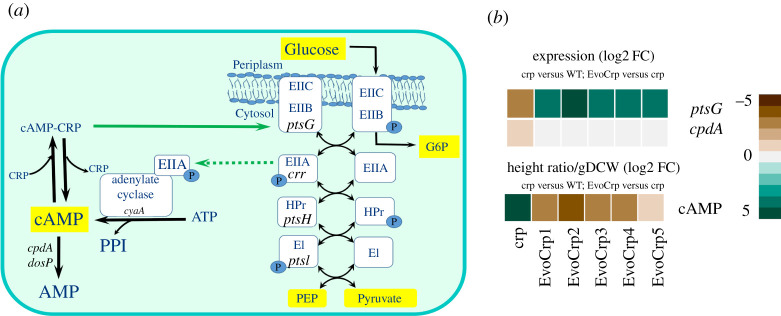
The fate of cAMP in the strains. (*a*) Network diagram of PEP-PTS system for glucose uptake with a schematic representation of the regulation on cAMP by the enzymes of PEP-PTS. Key metabolites of the pathway are shown in yellow blocks. The green solid arrow indicates positive gene regulation from our study and the green dotted arrow indicates positive regulation of activity from literature evidence. (*b*) Expression profile of the DE genes of PEP-PTS with the metabolite levels of cAMP. Fold change values for gene expression and metabolite levels are obtained by comparing *Δcrp* versus WT and EvoCrp versus *Δcrp*. Expression values are obtained from the average of two biological replicates expressed as log2 FC. Metabolite levels are obtained from the average of three biological and two technical replicates expressed at height ratio/gDCW (log2 FC). G6P, glucose-6-phosphate; PEP, phosphoenolpyruvate.

Next, to evaluate the metabolite levels that mirror the shifts in growth profiles [63,64], we characterized several metabolites of central carbon metabolism in the mid-exponential phase of batch growth in WT, *Δcrp* and the evolved strains using ^13^C-labelled metabolomics (electronic supplementary material, file S1) and the statistically significant metabolites (FDR < 0.05) were represented as boxplots (electronic supplementary material, figure S9). We integrated the metabolite levels with its cognate gene expression profiles to unravel the strain-specific adjustments at key nodes of metabolic pathways. Modulation of glucose uptake in *E. coli* can be inferred from the pool size of the physiological signal molecule cAMP, an inducer of CRP activity (figure 4*a*). We measured the intracellular cAMP levels and found that the deletion of CRP leads to approximately 55-fold higher (adj-*p* < 10^−9^) accumulation of cAMP compared to the WT (figure 4*b*). Counterintuitively, we observed no change in *cyaA* gene expression, which generates cAMP from ATP, and is known to be activated by the phosphorylated EIIA (*crr*) component of the PTS system (figure 4*b*) [65–67]. The *cpdA* gene, responsible for degrading cAMP, had lower expression in *Δcrp* (figure 4*a*). After evolution, we observed an approximately 8-fold decrease in cAMP levels without associated changes in gene expression across all EvoCrp strains relative to *Δcrp* that indicated an evolutionary restoration of cAMP levels albeit inefficiently (electronic supplementary material, figure S10A). On the contrary, there were no significant changes in the ATP levels across all the strains (electronic supplementary material, figure S10B), which mostly agrees with the gene expression of the electron transport chain coupled to ATP synthesis (electronic supplementary material, file S1). Since the pool sizes of ATP and cAMP are vastly different in magnitude, we related the concentrations obtained in our study with the absolute concentrations reported for *E. coli* K-12 MG1655 strain under similar glucose respiratory conditions [68]. First, we estimated the average concentration (height ratio per gram dry cell weight (DCW)) for ATP and cAMP for each of the strains. Next, we normalized the cAMP level by its ATP level for each of the strains (electronic supplementary material, file S1). We assumed that 0.36% of ATP is used for the production of cAMP in the case of WT. Considering the changes in cAMP and ATP pools in the *Δcrp* and the evolved strains, we obtained that 20% and 3–5% of ATP were used for cAMP synthesis in the *Δcrp* and EvoCrp strains respectively (electronic supplementary material figure S10C, file S1), that might be suggestive of partial recovery of optimization of ATP usage in the EvoCrp strains.

**Figure 5.**
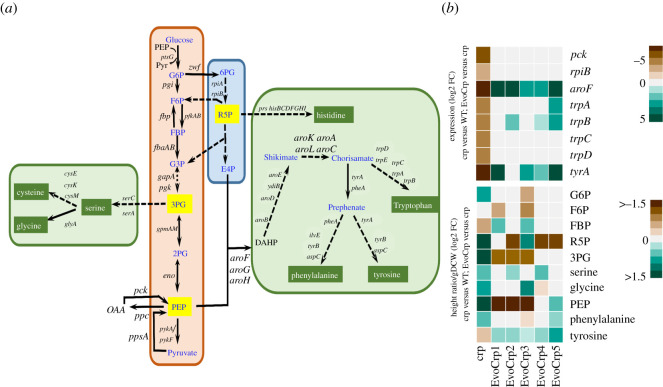
Integrated transcriptomics and metabolomics analysis at glycolytic and pentose phosphate pathway (PPP) nodes. (*a*) A network diagram depicting the glycolytic pathway, PPP and amino acid biosynthetic pathways generating from the precursor metabolites of glycolytic and PPP pathway. (*b*) Expression profile of DE genes and metabolite levels significantly altered in the glycolytic, PPP and amino acid biosynthetic pathways, were obtained by comparing *Δcrp* versus WT and EvoCrp versus *Δcrp*. Expression values are obtained from the average of two biological replicates expressed as log2 FC. Metabolite levels are obtained from the average of three biological and two technical replicates expressed as log2 FC of height ratio/gDCW. G6P, glucose-6-phosphate; PEP, phosphoenolpyruvate; F6P, fructose-6-phosphate; FBP, fructose 1,6-bisphosphate; 3PG, glycerate-3-phosphate; R5P, ribose-5-phosphate.

**Figure 6.**
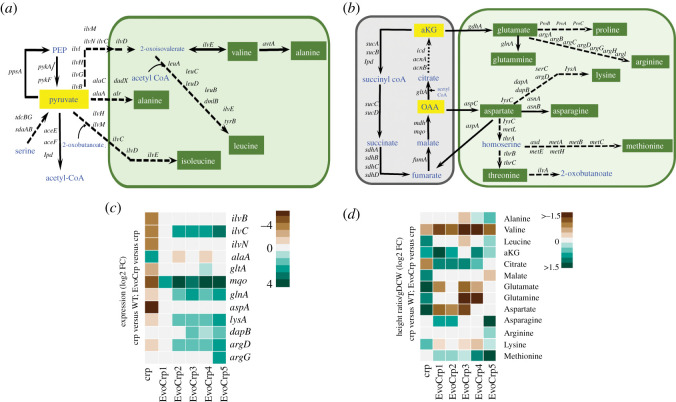
Integrated transcriptomics and metabolomics analysis at pyruvate and TCA cycle nodes. (*a*) A network diagram depicting branch points from pyruvate node and amino acid biosynthetic pathways generating from the precursor metabolite pyruvate. (*b*) A network diagram depicting branch points from TCA cycle and amino acid biosynthetic pathways generating from the precursor metabolites of TCA cycle. (*c*) Expression profiles of DE genes of pyruvate node, TCA cycle and amino acid biosynthetic pathways were obtained by comparing *Δcrp* versus WT and EvoCrp versus *Δcrp*. Expression values are obtained from the average of two biological replicates expressed as log2 FC. (*d*) Metabolite levels perturbed at the pyruvate node, TCA cycle and amino acid biosynthetic pathways, were obtained by comparing *Δcrp* versus WT and EvoCrp versus *Δcrp*. Metabolite levels are obtained from the average of three biological and two technical replicates expressed as log2 FC of height ratio per g DCW. PEP, phosphoenolpyruvate; aKG, α-ketoglutarate; OAA, oxaloacetate.

The gene expression pattern of *ptsG* (figure 4*b*) in EvoCrp strains corroborates with the nature of mutations in the promoter of the *ptsG* gene. Since glucose uptake in *E. coli* involves group translocation with the donation of phosphate from phosphoenolpyruvate (PEP) (figure 4*a*), we monitored the PEP levels [65,69]. We observed an increase in PEP concentration (aFC ∼ 8-fold) in *Δcrp* compared to the WT (figure 5*b*; electronic supplementary material, figure S9). Recovery of PEP levels was observed across the majority of the Evo*Crp* strains (aFC ∼ 6-fold, versus *Δcrp*). Akin to PEP, 3-phosphoglycerate (3PG) was found to be higher in *Δcrp* (aFC ∼ 3-fold, versus WT) and its level was reduced in EvoCrp strains (aFC ∼ 2-fold, versus *Δcrp*). Since increases in PEP and 3PG levels represent reliable indicators of carbon limitation [70], their recovery promptly sheds light on the restoration of carbon import. PEP is a precursor of the aromatic amino acids, namely phenylalanine, tyrosine and tryptophan. Concomitant with an increase in PEP levels, we observed a higher concentration of phenylalanine levels (aFC ∼1.4) in *Δcrp* (figure 5*b*; electronic supplementary material, figure S9). Conversely, gene expression of aromatic amino acid biosynthesis genes, namely *aroF* (aFC ∼ 19)*, tyrA* (aFC ∼ 13) and *trpAB* (aFC ∼ 3), showed significant downregulation in *Δcrp* mutant. Such antagonism highlighted the known negative feedback regulation of amino acid biosynthesis [71]. However, EvoCrp strains retained corresponding levels of phenylalanine compared to *Δcrp,* despite lowered levels of PEP and upregulation of *aroF* gene expression (aFC > 7). Pyruvate, an α-keto acid and end product of glycolysis, is a precursor of the branched-chain amino acids alanine, valine, leucine and isoleucine (figure 6*a*). We found a reduction in valine (aFC ∼ 1.5-fold) and an increase in leucine levels (aFC ∼ 2-fold) in *Δcrp* compared to WT. By contrast, we observed, reduction in valine (aFC ∼ 2.3-fold; figure 6*d*; electronic supplementary material, figure S9) and no significant changes in leucine in the EvoCrp strains compared to *Δcrp*.

We also determined the concentrations of citrate and other α-keto acids like alpha-ketoglutarate (*α*KG) and oxaloacetate (OAA), which are key intermediates of the TCA cycle (figure 6*b*). In *Δcrp*, citrate level was lower (aFC approx. 1.75) compared to WT, in agreement with the reduced gene expression of *gltA* (figure 6*c,d*), whereas its levels were restored after evolution despite no alteration in gene expression that might be attributed to increased TCA cycle activity in the EvoCrp strains [72]. We observed approximately 1.7-fold higher concentration of *α*KG in *Δcrp* compared to the WT as well as in EvoCrp compared to *Δcrp* (figure 6*d*; electronic supplementary material, figure S9). *α*KG accumulation has been known to indicate nitrogen limitation and is a measure of the anabolic functions of the organism [73]. Since *α*KG generation is associated with the synthesis of many proteinogenic amino acids [71], we speculate that the high intracellular levels of *α*KG in *Δcrp* as well as EvoCrp, might indicate either a possible scenario of nitrogen limitation or increased synthesis of amino acids to account for protein biomass. The *α*KG is a precursor for the amino acids glutamate, glutamine, arginine and proline. In *Δcrp* compared to WT, glutamate levels were upregulated (aFC ∼ 3), whereas in the EvoCrp strains compared to *Δcrp,* we observed reduced levels of glutamate (aFC ∼ 1.6). OAA, which can be interpreted from malate levels in the cell [74], serves as a precursor of amino acids like aspartate, asparagine, lysine, threonine and methionine levels (figure 6*b*). Concomitant with the higher OAA (inferred from malate, aFC ∼ 2-fold) levels, a ∼ 2.5-fold higher aspartate and ∼ 1.5-fold higher levels of lysine were seen in *Δcrp* mutant compared to WT (figure 6*d*; electronic supplementary material, figure S9). Despite no changes in OAA (inferred from malate) levels and approximately 1.75-fold reduction in aspartate concentration, we observed 1.6-fold higher asparagine and identical lysine levels in EvoCrp strains compared to *Δcrp*. Methionine, which showed no change in its concentration in *Δcrp* compared to the WT, was 1.6-fold higher in all the EvoCrp strains compared to its parent *Δcrp.* Thus, concomitant with changes in precursors, we observed changes in proteinogenic amino acids, which elucidated an inefficient utilization towards its cellular objectives or protein biomass.

### 2.5. Physiological characterization agrees with the underlying molecular mechanism that defines shifts in growth

**Figure 7.**
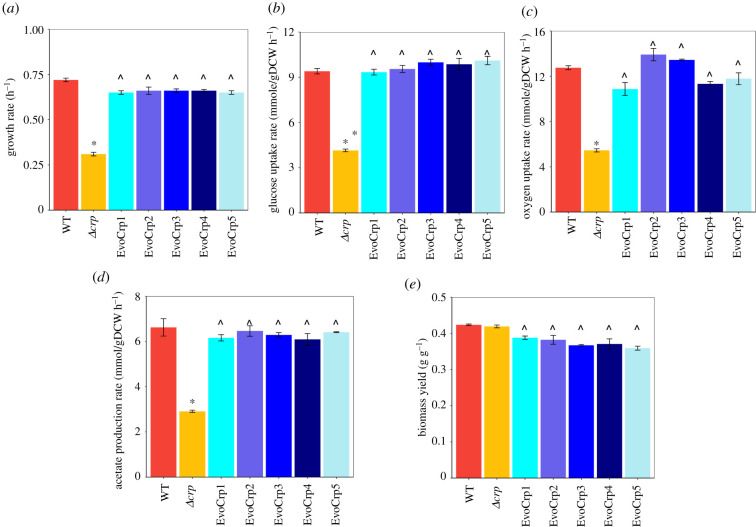
Phenotypic characteristics of the strains during batch exponential growth. Bar plot depicting the (*a*) growth rates, (*b*) glucose uptake rates (GUR), (*c*) oxygen uptake rates (OUR), (*d*) acetate production rates (APR) and (*e*) biomass yields of the WT, *Δcrp* and the EvoCrp strains. The error bars represent an average of growth rates obtained from three biological replicates. The error bars indicate the standard error across the replicates. The significance of the decrease in *Δcrp* versus WT is shown by an asterisk (*p* < 0.05, Student's *t*-test) whereas the significance of the increase in EvoCrp versus *Δcrp* is shown by a caret (*p* < 0.05, Student's *t*-test).

**Figure 8.**
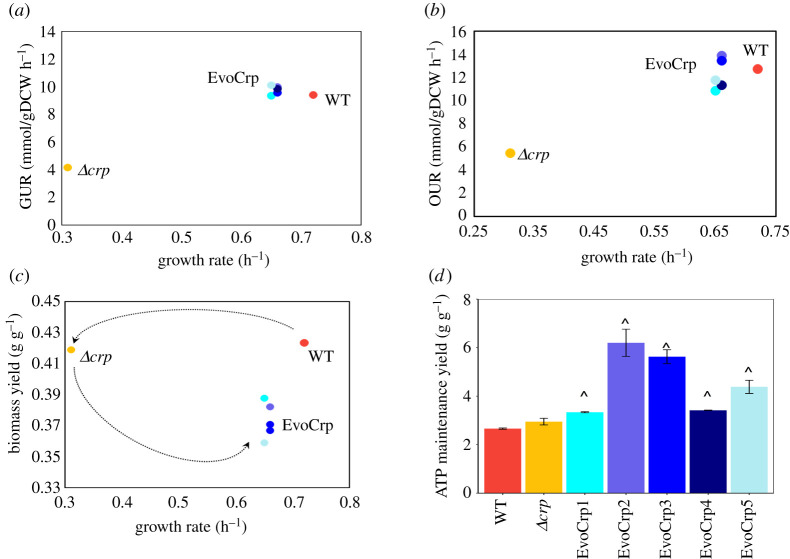
Pairwise correlation of phenotypic characteristics measured during batch exponential growth. (*a*) Pearson pairwise correlation between growth rates and glucose uptake rates (GUR) of all the strains (Pearson correlation coefficient, *r* = 0.96, *p* < 10^−3^). (*b*) Pearson pairwise correlation between growth rates and oxygen uptake rates (OUR) of all the strains (Pearson correlation coefficient, *r* = 0.92, *p* < 10^−2^). (*c*) Pearson pairwise correlation between growth rates and biomass yields (Pearson correlation coefficient, *r* = −0.87, *p*< 0.05, for EvoCrp and *Δcrp*). The dotted curves show the separation of the *Δcrp* and WT and migration of EvoCrp back towards WT as a result of evolution. (*d*) Bar plot depicting ATP maintenance (ATPM) yields expressed as (g/g) was predicted from flux balance analysis for WT, *Δcrp* and the EvoCrp strains using ATPM maximization as the objective function. The bars represent an average of the rate and yields obtained from three biological replicates. The error bars indicate the standard error across the replicates. The significance of the decrease in EvoCrp versus *Δcrp* is shown by a caret (*p* < 0.05, Student's *t*-test).

To evaluate how the changes in the transcriptome and the metabolome have directly impacted the phenotype of the organism, we characterized the growth rate, glucose uptake rate (GUR), acetate production rate (APR), oxygen uptake rate (OUR) and biomass yield (electronic supplementary material, file S1). It is to be noted that the maximum exponential growth rate was used as a metric to determine the growth fitness. We observed a marked reduction in growth rate (approx. 57%) in *Δcrp* strain compared to WT (figure 7*a*). The GUR and OUR in *Δcrp* were both significantly reduced by approximately 56%, compared to the WT (*p* < 0.05, Student's *t*-test) (figure 7*b,c*), which can be attributed to the reduction in gene expression of *ptsG* (figure 4*b*) and oxidative phosphorylation (electronic supplementary material, file S1), respectively. Similar trends were also observed in APR as well, despite no changes in its gene expression (figure 7*d*). Conversely, we observed an increase in growth rate (112%, *p* < 0.05; figure 7*a*) as well as GUR (approx. 130%, *p* < 0.05; figure 7*b*) and APR (approx. 115%, *p* < 0.05; figure 7*d*) in all EvoCrp strains compared to the *Δcrp*. The OUR in EvoCrp showed variability in its increase compared to *Δcrp* ranging from approximately 99% to approximately 155% (figure 7*c*). Further, we calculated the pairwise correlation of growth rate with GUR (Pearson correlation coefficient, *r* = 0.96, *p* < 10^−3^) and growth rate with OUR (Pearson correlation coefficient, *r* = 0.92) (figure 8*a,b*). This indicated that lowered GUR and lowered OUR strongly correlated with an overall reduction of growth rate in the *Δcrp* strain. In summary, our data suggested that all the parallel populations converged to phenotypes similar to WT at the end of ALE within approximately 100 generations.

The biomass yield for each of the strains was determined by normalizing the growth rates with their specific GURs. The WT and *Δcrp* strains showed similar biomass yields (approx. 0.42 g DCW per g glucose), thus indicating that loss of CRP did not perturb the carbon efficiency towards biomass synthesis in the mutant. However, there was a consistent decrease in the biomass yield (approx. 0.37 g DCW per g glucose) of EvoCrp strains that highlighted their inefficiency in optimally directing the carbon towards biomass (figure 7*e*). Correlation of the measured biomass yield and growth rate (Pearson correlation coefficient, *r* = −0.87, *p* < 0.05, for EvoCrp and *Δcrp*) showed the divergence of evolved strains away from *Δcrp* and WT (figure 8*c*). Since changes in biomass yields can be a consequence of alterations in ATP maintenance (ATPM, i.e. difference between the ATP production rate and its consumption rate towards biomass synthesis) [45,75], we predicted ATPM yields for each of the strains. Indeed, the EvoCrp strains had a significantly higher ATPM compared to the WT as well as *Δcrp*, which explained the allocation of carbon towards non-growth energy use. Besides, the accumulation of costly amino acids was in agreement with the higher unaccounted energy usage in the evolved strains (figure 8*d*). Overall, this phenomenon of lowered efficiency of carbon substrate allocation towards growth reinforced the rate–yield trade-off mechanism prevalent in ALE-adapted strains [76].

### 2.6. Model-based prediction of proteome allocation

**Figure 9.**
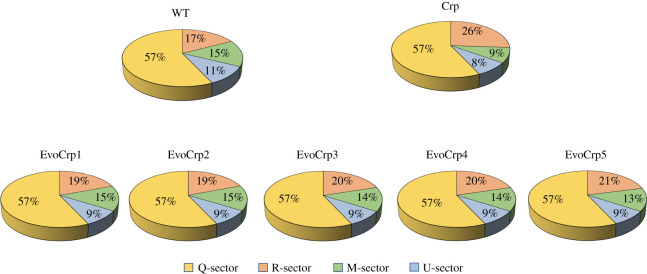
Proteome allocation. Changes observed in the percentage of proteome allocation towards R-,M- and U- sectors in WT, *Δcrp* and EvoCrp strains depicted as pie chart.

An inherent property of *E. coli* is to tightly coordinate the metabolism and protein economy in the cell towards an optimal resource allocation favouring growth fitness [34,63]. In the light of all the above observations, we sought to obtain insights into how necessary and unnecessary metabolic proteomes are affected, which directly associates with the shifts in growth rate. Towards this, we used the mathematical model based on the growth law theory to quantify the changes in proteome sectors as well as associate them with the changes in metabolite levels (electronic supplementary material, text S1). To account for the changes in the ribosomal sector (R-sector), we experimentally measured the R/P ratio (total RNA/total protein ratio) for each of the strains. Though indirect, the R/P ratio has been reported to be a quantitative agreement with the ribosome measurements from beta-galactosidase promoter studies as well as proteomics dataset [34,77,78]. We observed an increase in the R/P ratio in the *Δcrp* strain and a decrease in the R/P ratio in the EvoCrp strains (electronic supplementary material, file S1). Thus, the increase in R-sector is consistent with previous studies wherein strains faced with stress tend to increase their ribosome levels to hedge against unfavourable conditions [34,78]. However, as the investment for R-sector is expensive, the proteomic resources available for the metabolism become constrained. Next, we recalled a genome-scale ME-model (for metabolism and expression) that accounts for 80% of *E. coli* proteome [36,45,79,80], to assess how deletion of CRP and adaptive evolution affects the metabolic proteome allocation in the organism. The model predicted protein-coding genes along with transcript per million (TPM) calculations were employed to depict the necessary metabolic proteome (M-sector) and the unnecessary metabolic proteome (U-sector) fractions, specific to aerobic glucose metabolism in *E. coli* K-12 MG1655 (electronic supplementary material, file S1). Further, we used reported protein copies per cell and corresponding TPM values of representative genes to estimate the proteome fractions in all the strains, as described previously [81,82]. We observed a reduction in M-sector (catabolic and anabolic genes related to glucose metabolism) and an increase in U-sector (alternate carbon, glyoxylate shunt, osmotic stress-induced, amino acid degradation genes and chaperones) fraction in the *Δcrp* strain compared to WT that indicated a reduction in genes aligning with growth and increase in genes related to stress or hedging mechanisms, respectively (figure 9). Intuitively, our data revealed an upregulation in the M-sector fraction and downregulation in the U-sector fraction in EvoCrp strains to enhance their growth rate (figure 9). Further, a lower fraction of TCA cycle genes and secondary glucose transporters (*man* and *mal* genes) were found to be consistent across all the EvoCrp strains, to mitigate the unnecessary proteome cost towards their synthesis (electronic supplementary material, file S1).

The decrease in the M-sector and increase in U-sector and R-sector in *Δcrp* was reflected as increased *m*_E_/*t*_E_ ratio (metabolic efficiency/translation efficiency). The accumulation of amino acids and their precursors observed in *Δcrp* strain could be attributed to the large perturbation seen in the *m*_E_/*t*_E_ ratio (electronic supplementary material, file S1), implying an increase in their synthesis to meet the reduced translational capacity. On the contrary, the increase in M-sector and decrease in U-sector and R-sector in EvoCrp strains was reflected as restoration of the *m*_E_/*t*_E_ ratio towards the WT. However, the 10–35% higher *m_E_*/*t_E_* ratios observed in the EvoCrp strains compared to WT were mirrored as the accumulation of costly amino acids as well as TCA cycle metabolites like *α*KG and citrate. Overall, changes in metabolic and unnecessary metabolic proteome share towards biomass synthesis outline the trade-offs in proteome allocation, fundamental for balanced exponential growth [34].

## 3. Discussion

Adaptive mechanisms overarching genetic and metabolic regulatory networks are fundamental in conferring fitness advantages to strains when evolved in response to perturbations in their internal or external environments [37,45,46,48,83]. We systematically elucidated the pleiotropic changes due to the mutations in the *ptsG* promoter that enabled the rapid growth of the strains when evolved in the absence of CRP. Specifically, we report fine-tuning of proteome allocation, and corresponding metabolite adjustments such as rewiring of ATP towards the synthesis of costly amino acids away from the wasteful cAMP synthesis, in addition to enhanced rates of glucose uptake and related physiological traits that overall resulted in the increased growth rate of the evolved strains.

During evolution, mutations occurred predominantly in the *ptsG* promoter that generated additional ‘Pribnow-box’-like sequences that could potentially enhance the affinity of RNA polymerase sigma 70 towards the *ptsG* gene. Indeed, the lack of interplay of other regulators seen from *in vivo* binding studies and RNA polymerase sigma factor distribution seen from transcriptome analysis supports this basis. Notably, the mutations resulted in increased *ptsG* gene expression and thereby glucose uptake, emphasizing the role of *ptsG* promoter mutations in resolving the bottleneck caused by loss of CRP. Such a phenomenon was also observed in a previous study, thereby implying genetic parallelism across different *E. coli* sub-strains [43]. Despite such similarities, the difference in regulatory control of CRP on *ptsG* gene and the profound differences observed in genomic and phenomic states concur with the genetic background of the parent strains and the growth phase wherein the serial passages were carried out. Additionally, the absence of these mutations in aerobically evolved WT strains [36,55,56] highlighted the adaptive nature of the *ptsG* promoter mutations in response to the loss of CRP rather than adaptation to the media conditions.

Our findings demonstrated two principles of coordinated regulation: (i) the interplay between global regulators like CRP and global physiological factors on metabolism instrumental for the physiological growth status; and (ii) in the event of evolution, the mutations acquired together with growth-mediated effects become relevant to support the growth regimes of the fast-growing strains. We deconvoluted the complexity of gene expression by quantifying the extent of CRP regulation-specific effects and growth-mediated effects on the dysregulated metabolic pathways. Regardless of the glucose (excess glucose in batch versus glucose-limited chemostat) and growth conditions, we observed similar patterns in gene expression favouring stress-related or hedging functions over growth functions. We extended the quantification to the evolved populations as well to decouple the growth-mediated effects from mutation-specific effects. Our data not only underscored how evolution enabled the rewiring of the metabolic pathways, but also dissected the significant role of *ptsG* mutations in regulating these metabolic pathways from the prominent fast growth-mediated effects. Overall, we elucidated that the trade-off existing between the expression of growth-related genes (genes necessary for glucose metabolism) and the stress- or hedging-responsive genes determines the physiological outcome of the organism. The stress functions that were perturbed could be attributed to the chaperone-protein folding genes, genes responding to osmotic stress as well as amino acid degradation genes. Amino acid degradation genes are known to enable the organism to scavenge against nitrogen limitation or survive under acid stress conditions [62]. For instance, in *Δcrp*, these genes were found to be upregulated and the growth-related genes were downregulated, whereas in the evolved strains these stress-related genes were reverted to the WT state and growth-related genes were enhanced to facilitate their faster growth. Moreover, a similar pattern of gene expression was evident in the event of the introduction of a single point mutation in the *ptsG* promoter region in a strain lacking CRP (IG116-Δ*crp*). However, the substantial differences observed in its gene expression of metabolic pathways compared to the evolved populations can be attributed to the evolutionary resource adjustments as a result of enhanced growth fitness during ALE.

Previously, it was demonstrated that during carbon limitation, catabolic gene expression increases upon growth rate decrease, while during nitrogen limitation catabolic gene expression decreases upon a decrease in growth. The reverse was observed in the case of the anabolic gene expression. This response was shown to be coordinated by cAMP-CRP [31]. In our study, deletion of CRP entailed both carbon and nitrogen limitation as perceived from reduction in the GUR and the predicted ammonia uptake rate (electronic supplementary material, file S1) or *α*KG accumulation. Indeed, we observed reduced expression of the catabolic and the anabolic genes at a slower growth rate in agreement with the findings of You *et al*. [31]. Here, we not only emphasized the effects of CRP deletion but also demonstrated the effects of adaptive evolution on the proteome allocation of the organism. We developed a four-partition proteome model that integrates necessary metabolic genes with previously overlooked endogenous unnecessary metabolic genes. Our predicted system-wide proteome fraction of *Δcrp* mutant encompassing growth-mediated and CRP-driven effects highlighted that the absence of CRP resulted in disruptions of proteome allocation specifically as a reduction in necessary metabolic proteins (M-sector) and increase in unnecessary metabolic proteins (U-sector) compared to the WT. The M-sector, as inferred from theoretical proteome fractions of representative genes, comprised glucose uptake, catabolic and anabolic genes. Similarly, the U-sector represents the endogenous proteins entailed for stress (chaperone folding, amino acid degradation, osmotic stress) and hedging functions (alternate carbon metabolism, secondary glucose transporters) that are unnecessary under glucose metabolism. This reduced M-sector was further constrained by the increase in U-sector and R-sector. Recent studies have reported tight coordination of ribosomal protein expression with the growth rate of the organism [34,78]. Since the synthesis of ribosomes incurs a huge investment of proteomic resources [34,78], an increase in ribosome levels corresponds to an increase in unnecessary ribosomes to hedge for unfavourable conditions [78], thereby reducing the proteome share for metabolic proteins. Overall, CRP deletion perturbed metabolic and translational efficiency, which in turn resulted in alterations in growth rate changes that were reflected as the intracellular accumulation of amino acids and their precursor molecules. Apart from the known feedback regulation of these metabolites on the rate of amino acid biosynthesis [71], such accumulations can affect the carbon import flux [84], catabolic gene expression [31] as well as ribosomal levels [85]. This was evident from the increased accumulation of *α*KG, OAA (inferred from malate) and proteinogenic amino acids that were linearly correlated with the lowered GUR, reduced catabolic gene expression and increased ribosomal levels in *Δcrp*. The high intracellular levels of *α*KG in *Δcrp* also indicated a possible scenario of nitrogen limitation due to lowered ammonia uptake as indicated by the *in silico* flux prediction (electronic supplementary material, file S1). Further, *α*KG is known to be involved in the production of several amino acids which might suggest its anabolic functions to account for the protein biomass that was found to be consistent with the increased accumulation of amino acids observed in the strain. Thus, we showed that apart from carbon uptake, efficient proteome allocation between necessary and unnecessary metabolic proteome and associated metabolite adjustments are instrumental in facilitating optimal growth in an organism.

Evolved strains rebalanced the proteome by increasing the growth-promoting proteome M-sector and restoring the unnecessary U-sector and the R-sector towards the WT. Further, the evolved strains mitigated the proteome cost associated with the synthesis of expensive proteins such as enzymes of the TCA cycle and wasteful proteins such as proteins associated with secondary glucose transporters, alternate carbon metabolism and amino acid degradation. Such orchestration of the proteomic resources in the EvoCrp strains was reflected as restoration of the metabolic and translation efficiencies towards the WT state. Despite the restoration of their proteomic efficiencies, we still observed intracellular accumulations of several TCA cycle metabolites like *α*KG and costly amino acids that were not used for protein biomass. We suggest that the higher TCA cycle intermediates could be ascribable to a higher *in vivo* maximal rate of TCA cycle enzymes acquired during evolution, to meet the energetic demands of the cell [35]. In the EvoCrp strains, increased accumulation of *α*KG could be attributed to slightly higher glucose uptake and lowered ammonia uptake rates compared to the WT, resulting in an internal higher carbon/nitrogen ratio [absolute GUR (experimental)/absolute ammonia uptake rate (model-predicted)] (electronic supplementary material, file S1). As the organism perceives carbon excess to be a nitrogen-limited condition, we speculate the EvoCrp to be also facing nitrogen limitation indicated as *α*KG accumulation.

It is well known that growing cells maintain an optimal cAMP level necessary for proper carbon sensing [31] and hence ATP optimization. We observed a high accumulation of cAMP in the absence of CRP in conjunction with lowered GUR. On the contrary, evolved strains showed restoration of their cAMP levels, though inefficiently, mediated by reduced availability of phosphorylated PTS proteins for activation, with the net effect being partially alleviated ATP wastage. We speculate that this surplus ATP pool conserved from reduced cAMP synthesis was invested towards the synthesis of costly proteinogenic amino acids such as methionine, asparagine, lysine and arginine (considering only the number of activated phosphate bonds used in making the amino acid without the contribution of precursor synthesis itself) [86] that were found to accumulate in the evolved strains. This might be partially responsible for the higher ATPM yield, resulting in an overall reduction in the biomass yields observed in the evolved strains. Nevertheless, such inefficiencies involving excess carbon usage towards unaccounted-for energy reflect subpar utilization of carbon towards biomass in the evolved populations, which limits their ability to grow as energetically optimal as WT.

The current ALE study using a multi-omics approach has revealed mechanistic insights into the inherent systemic constraints that facilitate the final phenotypic response in conjunction with the selected mutations in the evolved strains to overcome the defects due to the loss of a global regulator. Despite perturbed proteome allocation towards necessary and unnecessary metabolic proteins in *Δcrp*, the carbon utilization efficiency towards biomass was not affected. On the contrary, we revealed that the evolved strains restored finely tuned proteome allocation that favoured growth over uneconomical hedging strategies and mitigation of costly proteome fractions at the expense of reduced carbon utilization efficiency. Our findings, based on proteome allocation in parent and evolved strains, bear striking similarities with proteome sector changes, representing a corollary to resource allocation defined by growth laws [34,85]. Future studies characterizing the genotype-to-phenotype relationship using such a multi-omics approach would expedite our understanding of microbial evolution across diverse conditions.

## 4. Material and methods

### 4.1. Strains

*Escherichia coli* K-12 MG1655 (CGSC#6300) was used as the parent strain in this study. All the other strains used in this study were derived from this strain (electronic supplementary material, file S1). We constructed *Δcrp* knockout in this genetic background by λ-Red mediated recombination [87], using plasmids pKD46, pKD13 and pCP20. After strain verification, glycerol stocks were made and stored at −80°C.

### 4.2. Physiological characterization in a bioreactor

For transcriptome, metabolome and phenotype characterizations, cells were grown in 500 ml bioreactor (Applikon) (planktonic state, batch culture) containing 200 ml M9 media (6 g/L anhydrous Na_2_HPO_4_, 3 g l^–1^ KH_2_PO_4_, 1 g l^–1^ NH_4_Cl, 0.5 g l^–1^ NaCl + 2 mM MgSO_4_ + 0.1 mM CaCl_2_), with 2g l^–1^ glucose and 40 mM MOPS. Briefly, cells from glycerol stocks were plated out on LB agar plate and a single colony was inoculated in LB media. A fixed volume of 100 µl cells was used to inoculate 50 ml preculture M9 + 40 mM MOPS media with 4 g l^–1^ glucose which was grown overnight in a shake flask at 200 rpm in a 37°C incubator (Eppendorf). The preculture cells, while still in exponential phase, were centrifuged and washed with M9, and inoculated in a bioreactor containing 200 ml M9 media with 2 g l^–1^ glucose such that the start optical density (OD) of all the cultures were ∼ 0.05 OD. The temperature of the bioreactor was maintained at 37°C and the pH of the media was maintained at pH 7.2 using 40 mM MOPS buffer to prevent the effects of pH change on growth. Aeration was done by sparging air in the bioreactor at 700 ml min^–1^ and at all times the dissolved oxygen (DO) levels were maintained above 40% saturation using mass flow controllers. Growth was monitored by collecting samples at regular intervals and measuring the OD at 600 nm in a spectrophotometer (Thermo Multiskan GO) until the organism reached its stationary phase. The growth rate was calculated from the slope of the linear regression line fit to ln (OD at 600 nm) versus time (in hrs) plot in the exponential phase. The DCW was determined from the OD at 600 nm values by using the experimentally derived relationship that 1.0 OD at 600 nm corresponds to 0.44 g DCW h^−1^. The phenotypic characterizations were done for three biological replicates (*n* = 3) across the exponential phase. The transcriptome and metabolomics characterizations were performed in the mid-exponential phase (0.6–0.7 OD). Samples were also collected during regular intervals to determine the rate of glucose uptake as well as the rate of secretion of extracellular metabolites. The samples were centrifuged and the supernatant was collected which was then used to determine the concentrations and rates using HPLC (Agilent 1200 Series) equipped with Bio-Rad Aminex HPX-87H ion exclusion column with 5 mM H_2_SO_4_ as the mobile phase. The column was maintained at a temperature of 50°C and the flow rate at 0.6 ml min^–1^. The specific rates were calculated from the change in substrate concentration over time, normalized to the biomass of each strain [88]. The biomass yield for each of the strains was determined by normalizing with their specific GUR (g DCW per g glucose). OUR was measured using a DO probe (Applikon) while growing the cultures in the bioreactor during its exponential phase.

For chemostat cultivations, the composition of the feed media was identical to the batch media. For all the strains, the cells were grown in batch condition until 80% of maximum biomass was obtained (as monitored by OD measurements) or until 80% of the glucose was consumed (as monitored by HPLC) after which the addition of feed media was started. The dilution rate was set to 0.21 h^−1^. After the cells reached the steady-state as indicated by OD measurements, cells were harvested after 3–5 residence times for transcriptomics analysis [45]. All physiological measurements were checked for statistical significance using unpaired two-tailed Student's t-test.

### 4.3. Adaptive laboratory evolution protocol

Adaptive evolution of replicate populations of *Δcrp* was carried out in shake flasks (planktonic state, batch culture) with M9 minimal media with 2 g l^–1^ glucose and 40 mM MOPS at 37°C. MOPS was added to maintain the populations at a constant pH during evolution (electronic supplementary material, figure S1A). All the replicate cultures were passaged serially into fresh media strictly in the mid-exponential phase to ensure that fitness gains occur primarily via increased exponential growth rates. This also prevents the cultures from entering the glucose-limited stationary phase and thereby avoids complexities associated with the onset of the stationary phase. As the growth rate of the organism changed during evolution, the volume of culture passaged was adjusted to prevent entry into the stationary phase. Glycerol stocks were made during each passage and were PCR verified for WT contamination using specific primers for the *crp* gene. This procedure was followed for 10 days (approx. 100 generations) and after it had reached a stable growth and no further increase in growth rate was observed, ALE was terminated. Genotype and phenotype characterizations of the evolved replicates were done for the population and not for individual clonal samples to ensure that all the traits of the population are taken into consideration. Characterization at the population level is a more efficient and feasible approach to understand the underlying mechanisms of evolution in an unbiased manner as it better reflects the properties of the population as a whole [89].

### 4.4. Whole genome resequencing

Genomic DNA from all the endpoint populations was extracted using GenElute Bacterial Genomic DNA Kit (NA2120; Sigma-Aldrich, St. Louis, MO) using the manufacturer's protocol. The integrity of the extracted genomic DNA was analysed by running it on an agarose gel and the quality was assessed and quantified using Multiskan GO (Thermo Scientific). The genomic DNA library was prepared using Illumina TruSeq DNA Nano Kit. The quality of the libraries was checked using Agilent Bioanalyzer. The libraries were then sequenced from both ends (paired-end) on Illumina HiSeq250 platform with 2 × 100 cycles. All the samples had an average of 275× mapped coverage.

The raw reads obtained from the sequencer were trimmed using CUTADAPT to remove TruSeq adapter sequences. The breseq pipeline [90] was used to identify the point mutations (SNPs), insertions and deletions mapped to *E. coli* K-12 MG1655 genome (GenBank accession no. NC_000913.3). Breseq was run using the -p option (for population samples) with default parameters to identify mutations present in the population at a frequency of less than 100%. Mutations that had 100% frequency were assumed to be mutations present in the WT strain and were not considered. Only the mutations predicted with high confidence under the category ‘predicted mutations’ were further analysed in this study.

### 4.5. Mutation validation

To determine the causality of the mutations detected by WGS, the mutations were introduced into the *Δcrp* background using *in vivo* site-directed mutagenesis [91]. However, to limit the scale of this study and given the consistent occurrence of mutations in the Fis binding region, only the IG116 mutation (SNPs) detected in the promoter of *ptsG* gene in the EvoCrp1 strain was selected for validation. Additionally, IG116 mutation was also investigated for Mlc binding. To construct the strains, scarless editing of the *E. coli* K-12 MG1655 *Δcrp* genome was done using a two-step recombination method using pSLTS plasmid (Addgene plasmid no. 59386). After the construction of the strains, they were sequenced to verify the correct introduction of the SNPs in the genome. This strain was further used to add 3X FLAG-tag to Fis protein or Mlc protein as described in the section below.

### 4.6. ChIP-qPCR

A 3X FLAG-tag was added to the C-terminus of the Fis or Mlc protein using pSUB11 plasmid [92] as the amplification template. The amplified construct was then introduced into *E. coli* K-12 MG1655 WT, *Δcrp* and IG116-Δ*crp* strain. The constructed strains were verified by PCR and Sanger sequencing. ChIP experiment was carried out using the protocol as described previously [93]. The DNA samples immune-precipitated by this method were recovered by PCR purification and were quantified by qPCR using primers for the specific as well as non-specific region (*frr*). Fold change in immunoprecipitated (IP-ed) DNA compared to mock DNA was calculated as 2^−ΔΔCt^ as described previously [94].

### 4.7. RNA extraction and enrichment of mRNA

For each strain, RNA extraction of two biological replicates was performed. The cells were grown till the mid-exponential phase and then 50 ml cells were harvested by centrifugation. RNA extraction was done using the TRIzol (Invitrogen)-chloroform method as previously described [44,95]. DNase treatment was done to remove any DNA contamination after which enrichment for mRNA was done using the MicrobExpress Kit (Invitrogen) following the manufacturer's protocol. Single-end, strand-specific libraries for RNA sequencing were prepared using NEBNext Ultra II Directional RNA library kit (New England Biolabs). The quantity of the mRNA was assessed using Multiscan GO (Thermo Scientific) Nanodrop and the quality, as well as integrity, was checked using BioAnalyzer. The sequencing was carried out on HiSeq 2500 Rapid Run Mode using a 1 × 50 bp format.

### 4.8. Transcriptome data analysis

The raw transcriptome files from batch mid-exponential and chemostat conditions were first trimmed using CUTADAPT to remove adapter sequences and low-quality reads. The reads were then mapped to the *E. coli* K-12 MG1655 genome (GenBank accession no. NC_000913.3) using BWA [96]. The sam file generated was then converted to compressed bam format using Samtools [97]. FeatureCounts [98] was used to assign counts at the gene level using the reference genome provided in the GTF format. Ecocyc database [99] (v. 21.5) was used to retrieve the annotations for 4466 genes. It is to be noted that rRNA, tRNA and sRNA genes were excluded from the analyses. Differential gene expression was analysed from the raw counts by EdgeR [100] after removing genes having less than 10 reads. The genes that showed ≥ 2-fold change in expression (in both directions) and had adjusted *p* < 0.05 (Benjamini–Hochberg) were considered as DE genes and used for further analysis. The DE genes of the strains from both experimental conditions were enriched for metabolic pathways using KEGG pathway classification [101] as defined in Proteomaps (www.proteomaps.net). The mapped genes were represented as Voronoi treemaps (v. 2.0) [102]. The significance of the upregulated and downregulated genes within each category was validated using a hypergeometric test (*p* < 0.05) in R (R Core Team 2019). For each up/downregulated category, we arbitrarily chose atleast five DE genes enriched to be considered for significance analysis. Those DE genes which do not have an assigned ‘Accession ID’ (Ecocyc V. 21.5) such as phantom genes and *crp* gene itself were excluded from the above analysis.

For sigma factor enrichment analysis, the KEGG pathway enriched DE genes obtained under batch mid-exponential conditions for the strains were further assessed based on gene targets of sigma factors using data available in EcoCyc and RegulonDB [103]. The upregulated and downregulated genes belonging to each regulator were then tested for significance using a hypergeometric test in R (*p*-value < 0.01). Only those sigma factors which regulated atleast 10% of the KEGG enriched upregulated and downregulated DE genes, were retained for this over-representation analysis. Pearson correlation of log2 fold changes of EvoCrp versus WT and *Δcrp* versus WT and statistical significance were performed in R (R Core Team 2019). For comparison of point mutation strain (IG116-Δ*crp*) with EvoCrp1, the transcriptome of IG116-Δ*crp* and EvoCrp1 was analysed with respect to WT and *Δcrp* independently.

For transcriptome analysis of WT and *Δcrp* from glucose-limited chemostat cultivations, the DE genes were annotated as ‘CRP-specific’ (electronic supplementary material, file S1). These represent genes that are not altered due to slow growth. These genes were then used to identify the CRP regulation-specific genes in the transcriptome changes of *Δcrp* compared to WT in batch mid-exponential phase. For identification of mutation-specific genes in EvoCrp strains, transcriptome analysis was carried out for two EvoCrp strains (EvoCrp1 and EvoCrp3) in comparison to *Δcrp* from glucose-limited chemostat cultivations. These represent genes that are not altered due to the faster growth of the EvoCrp strains. These genes from both the EvoCrp1 and EvoCrp3 chemostat cultivations were then used together to identify the mutation-specific genes in the transcriptome changes of all the EvoCrp strains compared to *Δcrp* in batch mid-exponential phase. To characterize the mutation-specific effects in the transcriptome of IG116-Δ*crp* strain compared to *Δcrp* in batch mid-exponential phase, the differentially expressed genes annotated as ‘CRP-specific’ from transcriptome analysis of WT and *Δcrp* from glucose-limited chemostat cultivations, were used. In the heatmaps generated based on log2 fold change in gene expression, only those genes with FDR less than 0.05 and 2-fold change in at least one condition were retained for comparative analysis (the same gene with FDR > 0.1 in other conditions were not considered).

### 4.9. Metabolomics

#### 4.9.1. Chemicals for metabolomics

LC-MS grade methanol, acetonitrile and ammonium hydroxide (≥25% in water) were purchased from Honeywell. Analytical grade chloroform, HPLC-grade water and LC-MS grade ammonium acetate were purchased from Sigma. All metabolites used as external standards were purchased from Sigma. Uniformly labelled U-^13^C (>99% purity) glucose was purchased from Cambridge Isotope Laboratories.

#### 4.9.2. Extraction of metabolites

Metabolite samples were harvested in the mid-exponential phase in biological triplicates and technical duplicates. A fast-cooling method [104,105] was used to quench the harvested cells as reported previously. Briefly, approximately 10 ml culture (∼ 6–7 OD cells) was directly poured into 2 ml chilled (4°C) M9 (without glucose) in a 50 ml falcon tube. The tube was then dipped in liquid nitrogen for 10 s to bring down the sample temperature to 0°C. To prevent the formation of ice crystals, the sample was vigorously agitated with the help of a digital thermometer. Samples were then immediately centrifuged at 0°C, 7800 rpm for 5 min. The supernatant was discarded and the pellet was snap-frozen in liquid nitrogen and stored at −80°C until metabolite extraction was done.

For extraction of metabolites, the sample pellet was dissolved in 700 µl chilled (−80°C) methanol: 300 µl chilled (−20°C) chloroform [105], followed by snap freezing in liquid nitrogen and homogenization using a hand-held pestle (Sigma no. Z359971) all within 2 min. ^13^C-labelled extracts as internal standards were generated for the quantification of key metabolite pool sizes using an isotope-based dilution method [106]. The samples were spiked at the earliest stage of extraction with a fixed volume of internal standard taken from the same batch. The sample tubes were then placed overnight on a Thermo-shaker maintained at 0°C and 400 rpm. To the sample tubes, 500 µl of 2% ammonium hydroxide (4°C) prepared in HPLC-grade water was added and incubated on ice for 10 min to enable phase separation. The tubes were then centrifuged at 4°C, 13 000 rpm for 15 min, and the aqueous layer was collected in a chilled 1.5 ml tube. The samples were then completely dried in a vacuum concentrator and stored at −80°C. Before analysis in LC-MS/MS, samples were reconstituted in 100 µl chilled (−20°C) acetonitrile: buffered water (60 : 40, v/v), centrifuged at 4°C for 10 min, and the supernatant was transferred to pre-chilled glass vials. Buffered water consisted of 10 mM ammonium acetate, pH 9.23 adjusted with ammonium hydroxide prepared in HPLC-grade water [107]. The volume of the pooled internal standard was standardized such that the external standard and internal standard peak height differed less than 5-fold [68]. Metabolites were extracted from three biological and two technical replicates (*n* = 6).

#### 4.9.3. LC-MS/MS settings

The samples were analysed using a high-resolution mass spectrometer in Orbitrap Q Exactive Plus (Thermo) equipped with a SeQuant ZIC-pHILIC column (150 mm × 2.1 mm × 5-micron packing, Merck) and a ZIC-pHILIC guard column (20 mm × 2.1 mm × 5-micron packing, Merck) under alkaline mobile phase conditions with ESI ion source. The ESI was operated in positive (M + H)^+^ and negative (M – H)^−^ polarity switching mode. The spray voltage was set at 4.2 kV and 3.5 kV for the positive and negative modes respectively. The temperature was maintained at 300°C and 320°C for the ion transfer capillary (ITC) and probe heater, respectively. A heated electrospray ionization probe II (H-ESI-II) probe was used with the following tune parameters: sheath gas, 29; auxiliary gas, 7; sweep gas, 0; S-lens at 45 arbitrary units. A full scan range of 66.7 to 1000 *m/z* was applied for positive as well as negative modes and the spectrum data type was set to profile mode. The automatic gain control target was set at 1e6 with a resolution of 70 000 at 200 *m/z*. Before analysis, cleaning of the LC-MS system and ITC along with mass calibration was done for both positive and negative ESI polarities by using Thermo Calmix solution along with MS contaminants to take into account lower mass ranges. The signals of compounds 83.06037 *m/z* (2 × ACN + H) and 119.03498 *m/z* (2 × Acetate-H) were selected as lock masses for positive and negative modes respectively with lock mass tolerance of 5 ppm [108].

The mobile phase for chromatographic separation comprised non-polar phase A (acetonitrile: water mixed in the ratio 9 : 1, 10 mM ammonium acetate, pH 9.23 using ammonium hydroxide) and polar phase B (acetonitrile: water mixed in the ratio 1 : 9, 10 mM ammonium acetate, pH 9.23 ammonium hydroxide). A linear gradient with flow rate of 200 µl/min was set as follows: 0–1 min: 0% B, 1–32 min: 77.5% B, 32–36 min: 77.5% B to 100% B, 36–40 min: hold at 100% B, 40–50 min: 100% B to 0% B, 50–65 min: re-equilibration with 0% B [107]. An injection volume of 5 µl was used for all the samples and standards.

#### 4.9.4. Metabolomics data analysis

The data from the machine was processed using the software package Xcalibur 4.3 (Thermo Fisher Scientific) Quan Browser. A semi-quantitative analysis was performed using peak heights of precursor ions with a signal/noise (S/N) ratio of more than 3, a retention time window of less than 60 s, and less than 5 ppm mass error. The peak heights of the samples were normalized to the peak height of the internal standards to obtain a height ratio. Only those metabolites were retained for analysis, which had naturally occurring ^12^C peak height less than approximately 10% of the ^13^C-labelled peak height in the internal standard. MetaboAnalyst [109] was used for identifying statistically significant metabolites on biomass normalized and g-log-transformed metabolite concentrations. Missing value imputation was performed using SVD impute function before normalization. The concentration of metabolites is expressed as height ratio normalized to biomass (as height ratio/gDCW). Metabolite levels with false discovery rate (FDR) less than 0.05 and ≥ 1.2-fold change in concentrations (in both directions) were considered for further analysis. In the heatmaps generated based on log2 fold change of metabolites, only those metabolites with FDR < 0.05 in atleast one condition were retained for comparative analysis (the same metabolite with FDR > 0.1 in other conditions were not considered).

### 4.10. ME model simulation and proteome fraction estimation

The ME model [79,80] was simulated to generate a protein-coding gene list, by constraining the GURs in a range as reported previously [36]. Any gene predicted to be expressed in any of the 20 simulations was classified as ‘M-sector’; genes within the scope of the ME model but not expressed or having very low expression (low protein synthesis flux) are classified as ‘U-sector’. Next, only the DE genes in atleast one condition (i.e. *Δcrp* versus WT, EvoCrp versus *Δcrp,* and EvoCrp versus WT) were combined and enriched for M-sector and U-sector gene list. Genes encoding ribosome-affiliated proteins were not considered in this categorization. Next, the theoretical estimation of proteome fraction for all these genes was done from proteome mass calculation [82] and using previously reported protein copies per cell [81]. The proteome fractions were estimated assuming the TPM values yield the proteome fraction in WT and a constant mRNA to protein translation efficiency rate across the strains. Majorly, genes having reported protein copy per cell values were used to represent M and U-sectors. The calculation of missing protein copies per cell for genes was performed as described previously [82]. This was followed by summing the proteome fractions of all M-sector and U-sector genes. These fractions were depicted as percentages after calculations using the growth law equation. The U/M ratio was obtained by dividing the U-sector and M-sector proteome fractions (electronic supplementary material, file S1).

### 4.11. Total RNA and total protein (R/P) estimation

Untreated total RNA was extracted in the mid-exponential phase using TRIzol-chloroform as mentioned previously [33,44,95] from two biological replicates (*n* = 2). Total protein was extracted and estimated in the mid-exponential phase using a protocol described previously [31,33] from two biological replicates (*n* = 2). The R/P (μg/μg) was calculated from total RNA concentration (μg ml^−1^) and the total protein concentration (μg ml^−1^) after normalizing to its respective OD at 600 nm and gram (dry cell weight) conversion factor (0.44 mg ml^−1^).

### 4.12. Calculation of non-growth ATP maintenance

The experimentally measured growth rates, glucose uptake, acetate secretion and OURs were used as flux constraints in the iJO1366 metabolic model [110]. Constrained-based flux balance analysis was performed using COBRApy [111] by maximization of ATPM reaction flux. This predicted ATPM flux was normalized to glucose uptake flux to calculate the yield (g ATP per g glucose) for each of the strains. The predicted ammonia uptake rates were obtained from this flux distribution.

### 4.13. Promoter prediction analysis

Prediction of CRP binding sites was performed using the FIMO tool (5.4.1) from the MEME suite. The consensus motif used for the analysis was ‘WTBKBKVNNNNNNTMACANW’ where W is A/T, B is any base except A, K is G/T, V is any base except T, M is A/C and N is any of the four bases. The consensus motif was determined from the position-weight matrix from previously identified binding sites [9,10]. Statistically significant motifs (*p* < 0.01) were retained such that the binding sites were strictly within 250 bp upstream of the transcription start site of the target gene. Any motif found within the coding region of any gene was excluded from the analysis.

## Data accessibility

The WGS data presented in this study are available at NCBI BioProject (https://www.ncbi.nlm.nih.gov/bioproject/) under accession no. PRJNA637503. The RNA sequencing data and the processed files from this study are available at NCBI Geo (https://www.ncbi.nlm.nih.gov/geo/query/acc.cgi?acc) under accession no. GSE152214. The metabolomics data presented in this study are available at the NIH Common Fund's National Metabolomics Data Repository (NMDR) website, the Metabolomics Workbench, (https://www.metabolomicsworkbench.org), where it has been assigned Project ID PR000957. The data can be accessed directly via its Project DOI (10.21228/M80D7P).
