# Correction to ‘Global pleiotropic effects in adaptively evolved *Escherichia coli* lacking CRP reveal molecular mechanisms that define the growth physiology’

**DOI:** 10.1098/rsob.220087

**Published:** 2022-04-06

**Authors:** Ankita Pal, Mahesh S. Iyer, Sumana Srinivasan, Aswin Sai Narain Seshasayee, K. V. Venkatesh

*Open Biol.* **12**, 210206. (Published online 16 February 2022). (doi:10.1098/rsob.210206)

The originally published version of this paper showed the incorrect figure legends for figures 2 and 3. The corrected legends and their corresponding figures are as below:

**Figure 2.**
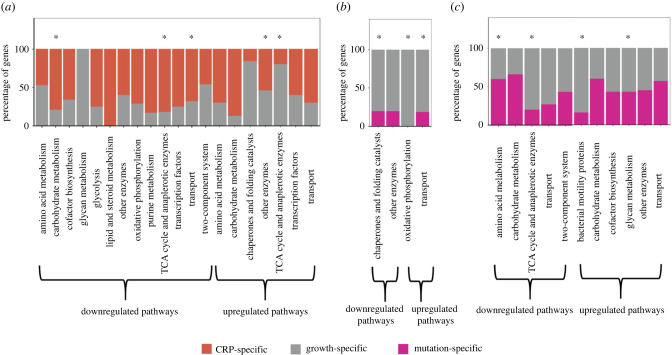
Dissecting the direct effects of CRP and mutation from the indirect effects of changes in growth rates. (*a*) The stacked plots showing the percentage of CRP-specific effects and growth-mediated effects in the genes of the downregulated and upregulated KEGG pathways in *Δcrp* versus WT. (*b*) The stacked plots showing the percentage of mutation-specific effects and growth-mediated effects in the genes of the downregulated and upregulated KEGG pathways in the EvoCrp strains (EvoCrp3 shown in figure) versus *Δcrp*. (*c*) The stacked plots showing the percentage of mutation-specific effects and growth-mediated effects in the genes of the downregulated and upregulated KEGG pathways in the IG116-*Δcrp* strain versus *Δcrp*. Significant pathways are denoted by asterisks (*p* < 0.05).

**Figure 3.**
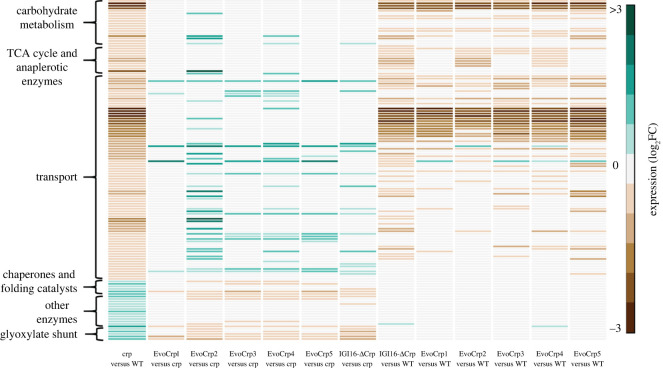
Comparative analysis of the KEGG pathway enriched genes across all the strains. Heatmap depicting the genes of the significantly altered pathways in *Δcrp* compared to WT. These pathways were also analysed in the EvoCrp strains and IG116-*Δcrp* strain compared to *Δcrp* and WT to identify the pattern of recovery in the gene expression.

